# Complete genome sequences of *Methylococcus capsulatus* (Norfolk) and *Methylocaldum szegediense* (Norfolk) isolated from a landfill methane biofilter

**DOI:** 10.1128/mra.00675-23

**Published:** 2024-01-18

**Authors:** David Pearce, Elliot Brooks, Charles Wright, Daniel Rankin, Andrew T. Crombie, J. Colin Murrell

**Affiliations:** 1 School of Environmental Sciences, University of East Anglia, Norwich, United Kingdom; 2 Norfolk County Council, Norfolk, United Kingdom; California State University San Marcos, USA

**Keywords:** methanotrophs, genomes, landfill, biofilter

## Abstract

Here we report the complete genome sequence of two moderately thermophilic methanotrophs isolated from a landfill methane biofilter, *Methylococcus capsulatus* (Norfolk) and *Methylocaldum szegediense* (Norfolk).

## ANNOUNCEMENT

The Strumpshaw closed landfill features a biofilter for the mitigation of the climate active gas methane, generated by the anaerobic breakdown of organic waste. This biofilter harnesses methanotrophic bacteria in a soil matrix for methane bio-oxidation. Two methanotrophs, *Methylococcus capsulatus* (Norfolk) and *Methylocaldum szegediense* (Norfolk), were isolated from this system. Biofilter soil was used to inoculate vials containing nitrate mineral salt (NMS) medium (1) and supplied with 20% (vol/vol) methane. Isolates were obtained from enrichment cultures by serial dilution and plating onto NMS agar plates, incubated in gas-tight containers supplied with 50% (vol/vol) methane. Optimal growth temperatures of the *Methylococcus* and *Methylocaldum* isolates were 45°C and 50°C, respectively. *M. capsulatus* (Norfolk) also grew on methanol (1%–5% vol/vol) as did *Methylococcus* strain MIR (2).

**Fig 1:**
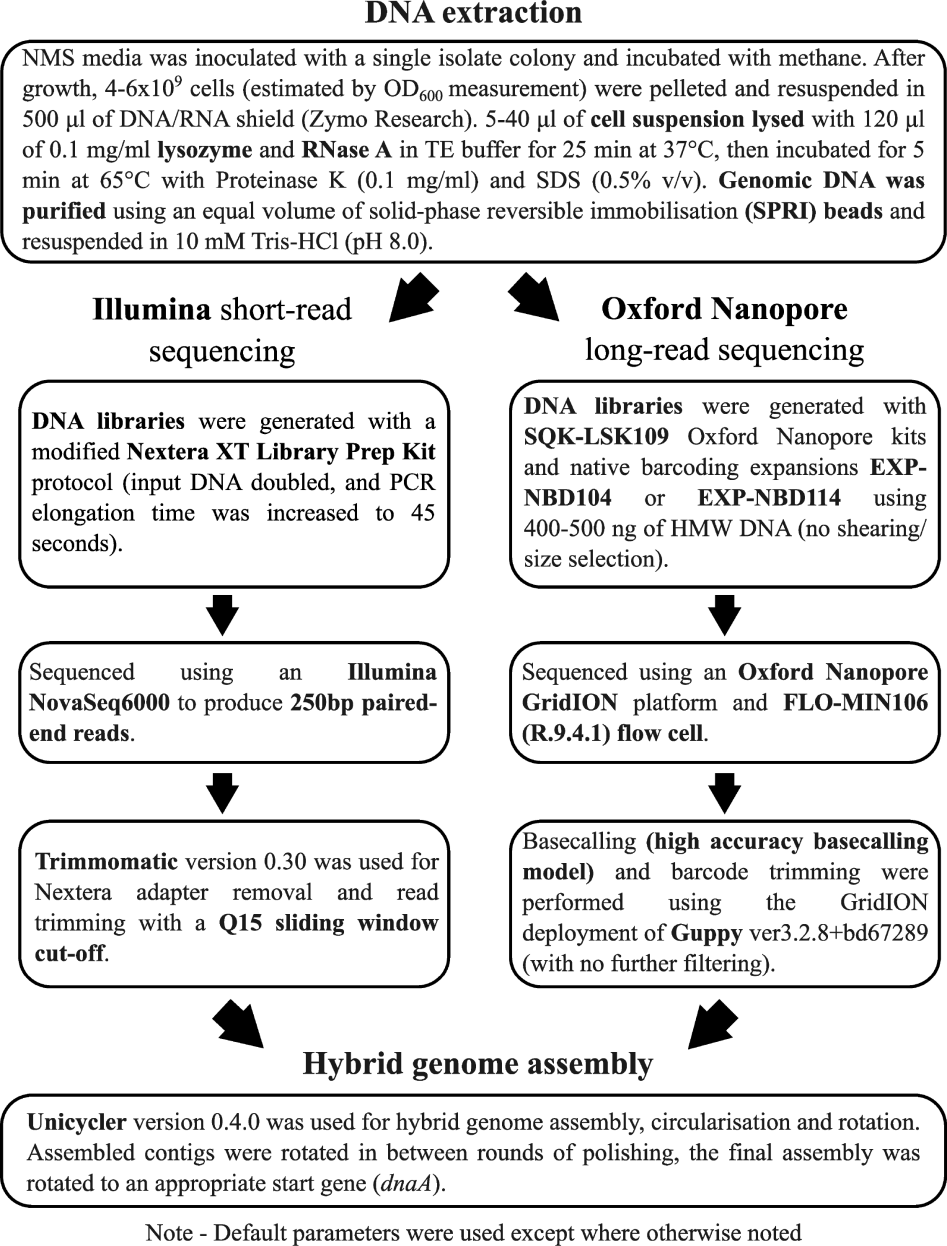
Sequencing and assembly pipeline.

DNA extraction, sequencing, and genome assembly were done using a combined long- and short-read sequencing service at MicrobesNG (Birmingham, UK) as described in Fig. 1. This pipeline was used to construct genomes for *M. capsulatus* (Norfolk) and *M. szegediense* (Norfolk), producing a closed genome in both cases.

MicroScope v.3.16.0 (3) was used for automated annotation and taxonomic assignment of assembled genomes before further manual curation. Genome assembly and sequencing read summaries are shown in Table 1.

**TABLE 1:** *Methylocaldum szegediense* (Norfolk) and *Methylococcus capsulatus* (Norfolk) genome summaries

DNA sequencing reads									
Isolate	Illumina total reads	Illumina read length (bp)		Nanopore total reads		Nanopore N_50_ (bp)		Illumina reads ENA accession no.	Nanopore reads ENA accession no.
*Methylocaldum*	936,436	250		184,537		4,370		ERR11151912	ERR11151913
*Methylococcus*	891,006	250		15,738		13,497		ERR11151914	ERR11151915

***Methylocaldum szegediense*** (Norfolk) assembly									
Replicon	Sequence length (bp)	Assembly coverage	% GC	No. of CDS	Ribosomal RNA genes			MicroScope assigned taxonomy	GenBank accession no.
					16S rRNA	23S rRNA	5S rRNA		
Chromosome	4,869,648	173×	57	5,038	2	2	2	*Methylocaldum szegediense*	GCA_949769195.1
Plasmid	25,724	481×	58	38	0	0	0		

***Methylococcus*** ***capsulatus*** (Norfolk) assembly									
Replicon	Sequence length (bp)	Assembly coverage	% GC	No. of CDS	Ribosomal RNA genes			MicroScope assigned taxonomy	GenBank accession no.
					16S rRNA	23S rRNA	5S rRNA		
Chromosome	3,398,174	85×	63.5	3318	2	2	2	*Methylococcus capsulatus*	GCA_949769275.1

The Norfolk isolates were assigned to the *Methylococcus capsulatus* and *Methylocaldum szegediense* spp. first described by Foster and Davis (4) and Bodrossy et al. (5). Based on average nucleotide identity (ANI) scores generated using CJ Bioscience’s online ANI calculator (6), the sequenced genomes with the highest similarity to *Methylococcus capsulatus* (Norfolk) and *Methylocaldum szegediense* (Norfolk) are *Methylococcus capsulatus* (Texas) (99.56%) and *Methylocaldum szegediense* (O-12) (99.64%), respectively (GenBank accession numbers GCA_000297615.1 and GCA_000427385.1).

Both genomes contain genes encoding a full methane oxidation pathway. Two *pmoCAB* clusters encoding particulate methane monooxygenase were found in each genome (7), and the *Methylococcus capsulatus* (Norfolk) genome also possesses a single soluble methane monooxygenase *mmoXYBZDCGQSR* cluster (8) and a putative copper chaperone (*mopE*) gene (9). Calcium-dependent (*mxaFJGIRSACKLD*) and lanthanide-dependent (*xoxFJ*) methanol dehydrogenase gene clusters (10, 11) were found in these genomes, with a clade 5 *xoxF* gene present in each and an additional clade 3 *xoxF* in *Methylocaldum szegediense* (Norfolk) (12). Both genomes feature complete gene inventories for tetrahydromethanopterin and tetrahydrofolate-linked formaldehyde oxidation, in addition to formate dehydrogenase genes (13). Carbon is presumed to be assimilated primarily via the ribulose monophosphate pathway as in *Methylococcus capsulatus* (Bath), although genes for a partial serine cycle and complete Calvin-Benson-Bassham pathway were detected (14). Alanine dehydrogenase and GS/GOGAT cycle genes for ammonia assimilation were present (15).

In addition to the 4.87 Mbp chromosome, *Methylocaldum szegediense* (Norfolk) also contained a ~25-kbp plasmid, encoding a plasmid replication initiator protein (TrfA), replication protein (RepA) and a toxin anti-toxin plasmid retention mechanism. A gene encoding a putative siphovirus Gp157 protein was also found, which may confer increased bacteriophage resistance (16, 17).

## Data Availability

Genome assembly and raw read accession numbers are listed in Table 1.
